# The whole-transcriptome landscape of muscle and adipose tissues reveals the ceRNA regulation network related to intramuscular fat deposition in yak

**DOI:** 10.1186/s12864-020-6757-z

**Published:** 2020-05-07

**Authors:** Hui Wang, Jincheng Zhong, Chengfu Zhang, Zhixin Chai, Hanwen Cao, Jikun Wang, Jiangjiang Zhu, Jiabo Wang, Qiumei Ji

**Affiliations:** 1 Key Laboratory of Qinghai-Tibetan Plateau Animal Genetic Resource Reservation and Utilization, Sichuan Province and Ministry of Education, Southwest Minzu University, Chengdu, Sichuan 610041 People’s Republic of China; 2 Qinghai-Tibetan Plateau Animal Genetic Resource Reservation and Utilization, Key Laboratory of Sichuan Province, Southwest Minzu University, Chengdu, Sichuan 610041 People’s Republic of China; 3 State Key Laboratory of Hulless Barley and Yak Germplasm Resources and Genetic Improvement, the Tibet Academy of Agricultural and Animal Husbandry Science , Lhasa, Tibet 850000 People’s Republic of China

**Keywords:** *Bos grunniens*, Intramuscular fat content, Transcriptome, Co-differentially expressed transcripts, ceRNA

## Abstract

**Background:**

The Intramuscular fat (IMF) content in meat products, which is positively correlated with meat quality, is an important trait considered by consumers. The regulation of IMF deposition is species specific. However, the IMF-deposition-related mRNA and non-coding RNA and their regulatory network in yak (*Bos grunniens*) remain unknown. High-throughput sequencing technology provides a powerful approach for analyzing the association between transcriptome-related differences and specific traits in animals. Thus, the whole transcriptomes of yak muscle and adipose tissues were screened and analyzed to elucidate the IMF deposition-related genes. The muscle tissues were used for IMF content measurements.

**Results:**

Significant differences were observed between the 0.5- and 2.5-year-old yaks. Several mRNAs, miRNAs, lncRNAs and circRNAs were generally expressed in both muscle and adipose tissues. Between the 0.5- and 2.5-year-old yaks, 149 mRNAs, 62 miRNAs, 4 lncRNAs, and 223 circRNAs were differentially expressed in muscle tissue, and 72 mRNAs, 15 miRNAs, 9 lncRNAs, and 211 circRNAs were differentially expressed in adipose tissue. KEGG annotation revelved that these differentially expressed genes were related to pathways that maintain normal biological functions of muscle and adipose tissues. Moreover, 16 mRNAs, 5 miRNAs, 3 lncRNAs, and 5 circRNAs were co-differentially expressed in both types of tissue. We suspected that these co-differentially expressed genes were involved in IMF-deposition in the yak. Additionally, *LPL*, *ACADL*, *SCD*, and *FASN*, which were previously shown to be associated with the IMF content, were identified in the competing endogenous RNA (ceRNA) regulatory network that was constructed on the basis of the IMF deposition-related genes. Three ceRNA subnetworks also revealed that *TCONS-00016416* and its target *SIRT1* “talk” to each other through the same miR-381-y and miR-208 response elements, whereas *TCONS-00061798* and its target *PRKCA*, and *TCONS-00084092* and its target *LPL* “talk” to each other through miR-122-x and miR-499-y response elements, respectively.

**Conclusion:**

Taken together, our results reveal the potential mRNA and noncoding RNAs involved in IMF deposition in the yak, providing a useful resource for further research on IMF deposition in this animal species.

## Background

The intramuscular fat (IMF) content in livestock is positively correlated with various aspects of meat quality, such as tenderness, flavor, and juiciness, and as such is one of the key traits related to consumer preference. The IMF refers to the sum of phospholipid, triglyceride, and cholesterol contents within muscles, and is considered as the last type of fat developed during fat deposition. Research has revealed that the IMF content is determined both by hypertrophy and hyperplasia of adipocytes during the development of livestock species [1]. The factors that related to the variation of IMF content in livestock include the species, breed, muscle types, gender, age, and nutrition level [2, 3]. Mechanisms such as nutrient regulation ultimately affect the deposition of IMF by affecting the transcription, mRNA expression, protein expression, and modification of genes. Studies have found the heritability of the IMF content to range from 0.47 to 0.53 [4–6]. However, because the IMF content can only be measured after animal slaughter, since there are no instruments that can measure it in vivo, it is difficult to improve this trait by the traditional selection methods. Hence, molecular breeding based on the mechanism of IMF metabolism is a key method used for IMF content improvement [7]. However, no effective marker for IMF content selection practices in the livestock has yet been found.

The yak (*Bos grunniens*), one of the ruminants that live in the Qinghai-Tibet Plateau and adjacent areas, is well adapted to the high-altitude environments. Compared with cattle meat, yak meat has higher contents of protein and mineral substance, but a lower content of fats, especially IMF [8]. A poor IMF deposition ability is a common phenomenon in yaks, and there are no known populations or breeds of yaks with an excellent IMF deposition ability. Therefore, to improve this ability of yaks fundamentally, the key genes affecting the molecular genetic mechanism of IMF deposition in this species need to be found.

The IMF content depends mainly on the size and number of intramuscular adipocytes and muscle growth rate [2], indicating that muscle cells and adipocytes interact with each other during IMF deposition. Both adipocytes and myocytes originate from mesenchymal stem cells [9, 10]. Moreover, the muscles and adipose tissue are considered as major endocrine organ that secrete numerous proteins, named myokines and adipokines, respectively [11, 12]. Myostatin, which is secreted from myocytes, decreases the IMF content by inhibiting the differentiation of preadipocytes [13]. It was reported that the coculture of C2C12 skeletal muscle cells with 3 T3-L1 adipocytes increased the gene expression of peroxisome proliferator-activating receptor gamma (*PPARγ*), fatty acid synthase (*FASN*), and fatty acid-binding protein (*FABP4*) [14], which interestingly are genes that play a key roles in fatty acid metabolism and have also been demonstrated to be related to IMF deposition [15–17]. These findings indicate that muscle cells are involved in the regulation of lipid-related factors in adipocytes and may participate in the IMF deposition processes. Many recent studies on the mechanism of IMF deposition in cattle have already revealed some of the genes that are involved in the IMF deposition-regulating pathway [18]. However, the genes associated with IMF deposition in yaks and their related molecular mechanisms remain unknown. The one-by-one identification of the potential regulatory genes in the yak would undoubtedly be like trying to find a needle in a haystack. Moreover, previous studies have showed that the IMF content varies even between breeds of the same species [19] and between different development stages [20]. Previous studies showed that the IMF content of *longissimus dorsi (LD)* in 0.5-year-old yaks were significantly lower than that in adult yaks [21], but was similar among adult yaks of different ages, which is unlike the situation in cattle where the IMF content of this same muscle increase with advancing age. Taken together, these results indicate that the regulation of MF deposition is species specific.

The yak used in this study are part of a dual-purpose (i.e., indigenous meat-dairy) population that is distributed in Changdu city, Tibet province, China. After long-term interbreeding, the yaks have attained consistency in appearance, reproductive and production performances. Until now, a global analysis of the molecular mechanism of IMF deposition in yak has not been previously performed. Therefore, the elucidation of the differences in the whole transcriptomes related to IMF deposition at different development stages of the yak is essential for interpreting the function of the DEGs. In this study, the IMF contents in 0.5-, 2.5-, 4.5-, and 7.5-year-old yaks were determined, and the whole-transcriptome profiles of the LD muscle and its adjacent intermuscular adipose tissues (AA) in the 0.5- and 2.5-year-old yaks were obtained to compare the DEGs in these two tissues between the two developmental stages. Then, the co-DEGs were obtained and considered as the DEGs involved in IMF deposition. Using clustering analysis and advanced visualization techniques, several genes and pathways involved in adipogenesis and lipogenesis were revealed. Finally, we constructed a comprehensive competing endogenous RNA (ceRNA) network on the basis of the co-DEGs between the LD and AA tissues to highlight the genes that are most likely to be involved with the IMF trait in yaks.

## Results

### Intramuscular fat contents of the *longissimus dorsi* muscle in yaks of different ages

**Fig 1:**
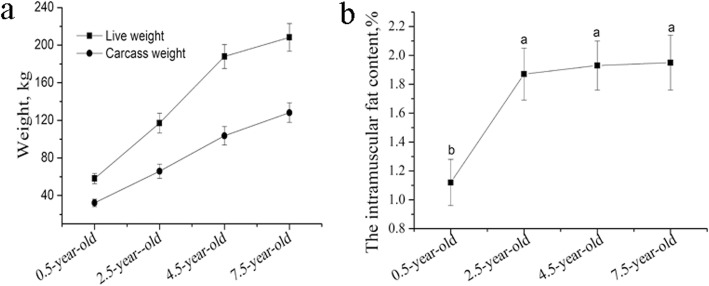
The dynamics in the live weight (**a**) and the intramuscular fat (IMF) content (**b**) across 0.5-, 2.5-, 4.5-, and 7.5-year-old of age.

The IMF content of the LD increased along with the development of the yaks from 0.5 to 7.5 years of age. Compared with the IMF content in the 0.5-year-old yaks, that in the 2.5-year-old animals was significantly higher (*p* < 0.05), and this age group also showed the fastest LD fat deposition of the yaks. However, the IMF content increased slightly from the 2.5-year-old to the 7.5-year-old animals (Fig. 1a and b),

### Overview of RNA sequencing

To assess the genes involved in IMF deposition, LD and AA tissues were collected from the 0.5- and 2.5-year-old yaks for the whole-transcriptome profiling of all mRNAs and noncoding RNAs (long noncoding RNAs (lncRNAs), circular RNAs (circRNAs), and microRNAs (miRNAs)) via high-throughput sequencing. For the RNA-sequencing (RNA-Seq) libraries, an average of 95.62 million clean reads were obtained from the 12 samples tested, and 87.91–90.13% of these reads were uniquely aligned to the reference genome Ensemble *BosGru* v2.0. All 12 samples had at least 94.80% reads equal to or exceeding Q30 (Table S1). In addition, for the small RNA-Seq libraries, an average of 10.80 million clean reads were obtained. An average of 9.59 million known miRNA reads, 1.57 thousand novel miRNA reads, and 28.21 thousand unannotated reads were obtained after a series of analyses (Table S2).

In total, 45,366 mRNAs (Table S3) were obtained, of which 84.88% were generally expressed in both LD and AA tissues. Moreover, 22,596 and 22,770 known and novel mRNAs were identified, respectively, which included 2737 LD tissue-specific and 4122 AA tissue-specific mRNAs (Table S4). Additionally, 4142 lncRNAs were obtained, of which 3600 and 3761 were expressed in the LD and AA tissues, respectively, and 77.72% were consistently expressed in both types of tissues. Of these lncRNAs, 383 were LD tissue specific and 541 were AA tissue specific (Table S4). Furthermore, 1444 miRNAs were identified, of which 1290 were known and 154 were novel (Table S3). Finally, 39,853 circRNAs were identified in the yak (Table S3), of which 17,211 and 9616 were found to be LD tissue specific and AA tissue specific, respectively (Table S4).

### Differentially expressed mRNAs during intramuscular fat deposition

**Fig. 2:**
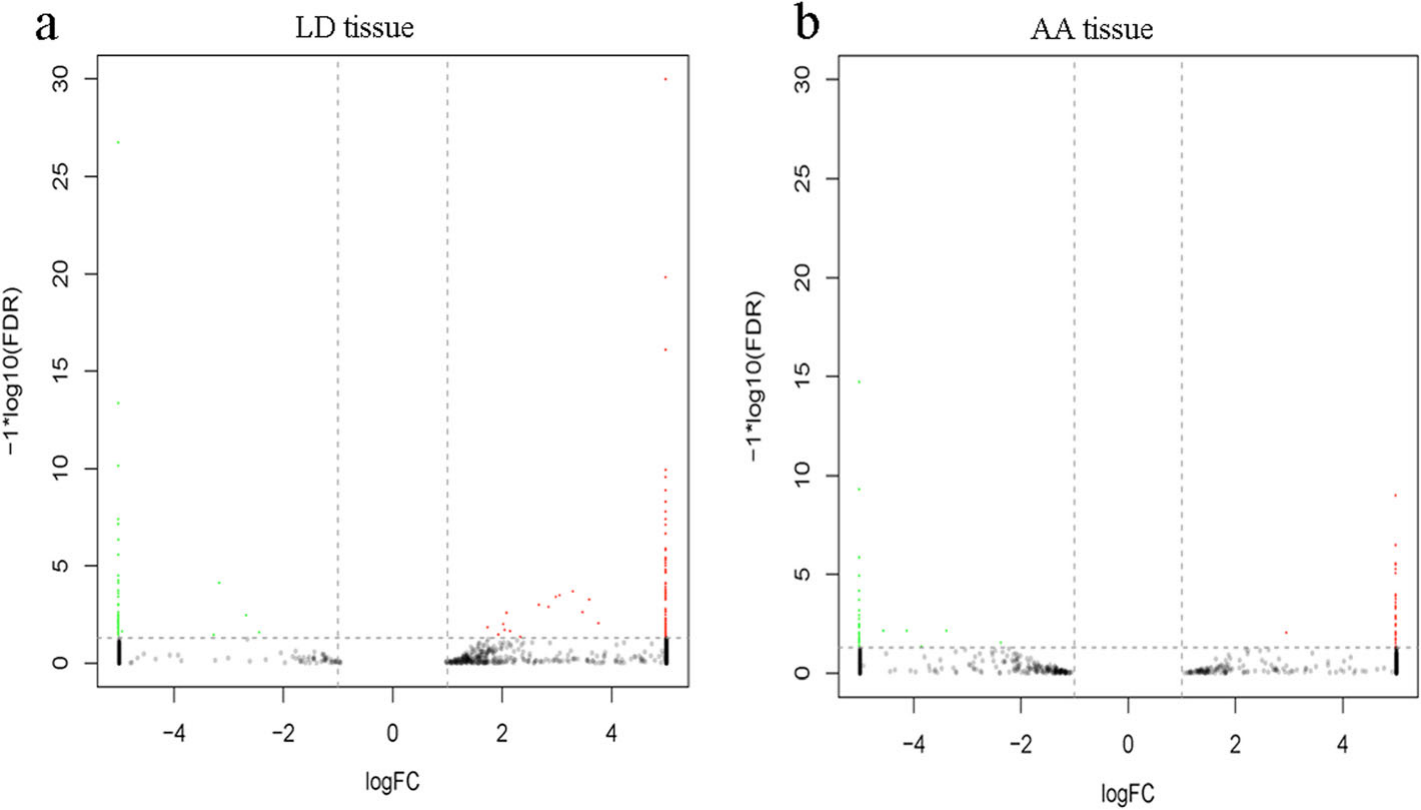
Differentially expressed mRNAs during LD (**a**) and AA (**b**) tissues development, respectively. The red dots and blue dots respectively represent up-regulated and down-regulated mRNAs during development

**Fig. 3:**
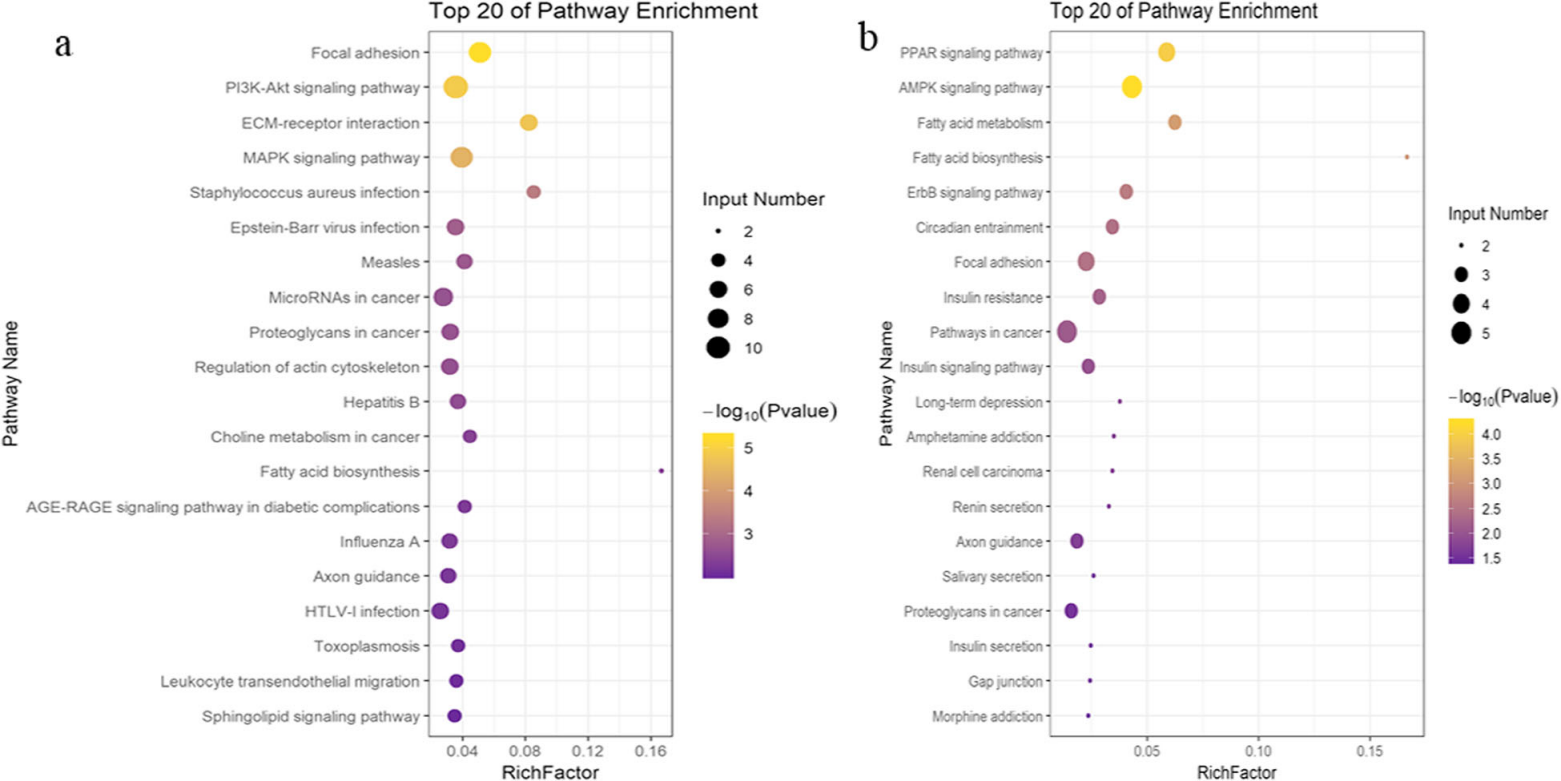
KEGG pathway analysis for differentially expressed mRNAs in LD (**a**) and AA (**b**), respectively. Only the top 20 enriched pathways are presented here

First, DEGs between the 0.5- and 2.5-year-old LD tissues were screened, whereupon 149 DEGs were found, of which 44 were downregulated and 105 were upregulated (Fig. 2a, Table S5), these DEGs included elongation of very-long-chain fatty acids 7 (*ELOVL7*, log2 fold change (FC) =10.377), long-chain acetyl-Coenzyme A dehydrogenase (*ACADL*, log2FC = 11.897), stearoyl-CoA desaturase (*SCD*, log2FC = 3.065), *FASN* (log2FC = 2.061) and sirtuin 1(*SIRT1*, log2FC = − 9.180), which are related to the regulation of triglyceride accumulation. Gene Ontology (GO) enrichment analysis revealed that these DEGs were involved in the positive regulation of histone methylation (GO:0031062), tissue morphogenesis (GO:0048729) and protein tyrosine kinase activity (GO:0004713). Moreover, these DEGs were also enriched in GO terms related to lipid metabolism, such as the lipid biosynthetic process (GO:0008610) and fatty acid biosynthetic process (GO:0006633) (Table S5). The Kyoto Encyclopedia of Genes and Genomes (KEGG) pathway analysis revelved that these DEGs were significantly enriched in phosphatidylinositol-3-kinase (PI3K)-protein kinase B (Akt) signaling pathway, focal adhesion, mitogen-activated protein kinase (MAPK) signaling pathway, and and extracellular matrix (ECM)–receptor interaction (Table S5, Fig. 3a).

Similarly, after comparison of the data between the 0.5- and 2.5-year-old AA tissues, 72 DEGs were obtained, of which 39 were upregulated and 33 were downregulated (Fig. 2b, Table S5). These included lipid metabolism related genes, such as sterol regulatory element binding transcription factor 1(*SREBF1*, log2FC = 6.173), *ACADL* (log2FC = 9.478), *ELOVL7* (log2FC = 8.814), *PPARγ* (log2FC = 6.996), and *SIRT1* (log2FC = − 9.299) (Table S5). GO analysis revealed that these DEGs were enriched for terms in lipid metabolism, such as cellular response to lipid (GO:0071396), fatty acid biosynthetic process (GO:0010885), regulation of cholesterol storage (GO:0010885), and response to lipid (GO:0033993) (Table S5). KEGG analysis revealed the top 5 pathways of these DEGs to be the AMP-activated protein kinase (AMPK) signaling, PPAR signaling, fatty acid metabolism, fatty acid biosynthesis, and ErbB signaling pathways (Table S5, Fig. 3b).

Furthermore, there were 16 DEGs in common in both the LD and AA comparison groups (Table 1); namely, acetyl-CoA carboxylase beta (*ACACB*), G protein subunit alpha 12 (*GNA12*), autism susceptibility candidate 2 (*AUTS2*), Xeroderma pigmentosum group A (XPA)-binding protein 2 (*XAB2*), *ACADL*, repulsive guidance molecule B (*RGMB*), SMAD family member 1(*SMAD1*), *ELOVL7*, *SIRT1*, *FASN*, protein kinase C alpha (*PRKCA*), mitogen-activated protein kinase kinase kinase kinase 1(*MAP 4 K1*), zinc finger protein 41(ZNF41), lipoprotein lipase (*LPL*), hypoxia inducible factor 1 subunit alpha (*HIF1A*), and *SCD*. These results indicated that these 16 DEGs may have a role in the regulation of IMF-deposition development.

**Table 1:** The co-differentially expressed genes between LD and AA tissues

ID	Differentially expressed genes in LD aging					Differentially expressed genes in AA aging					
	0.5-LD^a^	2.5-LD^b^	log2(FC)^c^	*P*-value	FDR	0.5-AA^d^	2.5-AA^e^	log2(FC)	P-value	FDR	Symbol
TCONS_00004555	0.840	0.001	−9.714	0.000	0.016	0.001	0.707	9.465	0.000	0.043	GNA12
TCONS_00014807	0.001	1.130	10.142	0.000	0.000	0.001	1.530	10.579	0.000	0.000	AUTS2
TCONS_00019781	0.001	1.383	10.434	0.000	0.005	1.040	0.001	−10.022	0.000	0.043	XAB2
TCONS_00055593	0.033	6.587	7.626	0.000	0.000	2.513	0.001	−11.295	0.000	0.017	RGMB
TCONS_00062820	0.001	0.987	9.946	0.000	0.016	29.180	2.817	−3.373	0.000	0.007	PRKCA
XM_005888988.2	0.001	3.813	11.897	0.000	0.000	0.001	0.713	9.478	0.000	0.000	ACADL
XM_005889058.2	0.001	1.330	10.377	0.000	0.000	0.001	0.450	8.814	0.000	0.002	ELOVL7
XM_005890693.1	0.001	0.337	8.396	0.000	0.029	3.397	0.001	−11.730	0.000	0.020	HIF1A
XM_005891509.1	0.953	0.001	−9.897	0.000	0.012	0.001	1.893	10.887	0.000	0.000	ACACB
XM_005892055.2	6.423	53.737	3.065	0.000	0.000	0.001	1.890	10.884	0.000	0.016	SCD
XM_005905364.2	16.140	67.330	2.060	0.000	0.019	1.030	8.033	2.963	0.000	0.008	FASN
XM_005906559.1	0.001	0.773	9.595	0.000	0.000	6.443	0.001	−12.654	0.000	0.000	SMAD1
XM_005907329.1	0.580	0.001	−9.179	0.000	0.000	0.630	0.001	−9.299	0.000	0.043	SIRT1
XM_005910150.2	0.001	2.177	11.088	0.000	0.000	1.360	0.001	−10.409	0.000	0.000	MAP 4 K1
XM_014479307.1	0.001	0.460	8.845	0.000	0.040	0.001	2.873	11.489	0.000	0.029	ZNF41
XM_014480300.1	0.001	0.737	9.525	0.000	0.005	338.727	19.580	−4.113	0.000	0.007	LPL

^a^0.5-LD: 0.5-year-old *longissimus dorsi* muscle tissues

^b^2.5-LD: 2.5-year-old *longissimus dorsi* tissues

^c^FC: FPKM fold change between different groups

^d^0.5-AA: 0.5-year-old adjacent adipose tissues

^e^2.5-AA: 2.5-year-old adjacent adipose tissues

### Total lncRNAs and differentially expressed lncRNAs during intramuscular fat deposition

To reveal the potential functions of the 4142 identified lncRNAs in IMF deposition, three independent algorithms—antisense (mRNA sequence complementarity), cis (genomic location), and trans (expression correlation) — were performed to predict the target genes of the lncRNAs. In total, 3963 target genes were predicted, of which 332 were targets of 421 antisense lncRNAs, 1089 were targets of 826 cis-acting lncRNAs, and 3214 (1487) showed the most positively (negatively) correlated co-expressed with 4142 *trans*-acting lncRNAs (Table S6). KEGG analysis revealed that the antisense lncRNAs were significantly enriched for glycolysis and gluconeogenesis pathways (Table S6), and the *trans*-acting lncRNAs were significantly annotated to pathways of lipid and carbohydrate metabolism, such as the steroid hormone biosynthesis, ascorbate and aldarate metabolism, and starch and sucrose metabolism pathways (Table S6). Additionally, even though they were not significantly enriched in any pathways, the *cis* acting lncRNAs were involved in the transforming growth factor-beta (TGF-β) signaling and Hedgebog signaling pathways, which play key roles in lipid metabolism (Table S6).

Four differentially expressed lncRNAs (DELs) (2 up-regulated and 2 down-regulated) and 9 DELs (8 up-regulated and 1 down-regulated) were identified in the LD and AA tissues, respectively (Table 2). As a preliminary exploration of the functional implications of the DELs across genomes, we investigated whether lncRNAs were co-regulated with the DEGs during IMF-deposition. Interestingly, in both LD and AA tissues, we observed that the antisense lncRNA *TCONS_00084092* targeted *LPL* as its differentially expressed co-target gene, whereas the two *trans*-acting lncRNAs *TCONS_00016416* and *TCONS_00061798* targeted *SIRT1* and *PRKCA*, respectively, as their differentially expressed co-target genes (Tables 2 and 3).

**Table 2:** Differentially expressed lncRNAs of 0.5-year-old LD vs 2.5-year-old LD

lncRNA ID	0.5-LD	2.5-LD	log2(FC)	*P*-value	FDR	Differentially expressed co-target
TCONS_00084092	0.001	1.753	10.776	0.000	0.000	LPL
TCONS_00022486	1.237	0.001	−10.272	0.000	0.000	/
TCONS_00016416	0.020	0.460	4.524	0.000	0.003	SIRT1
TCONS_00061798	0.127	0.001	−6.985	0.000	0.003	PRKCA

**Table 3:** Differentially expressed lncRNAs of 0.5-year-old AA vs 2.5-year-old AA

lncRNA ID	0.5-AA	2.5-AA	log2(FC)	*P*-value	FDR	Differentially expressed co-target
TCONS_00006234	0.003	0.650	7.607	0.000	0.022	/
TCONS_00016416	0.010	0.542	5.76	0.000	0.044	SIRT1
TCONS_00019146	0.001	0.240	7.907	0.000	0.036	/
TCONS_00084092	0.001	0.317	8.306	0.000	0.047	LPL
TCONS_00031879	0.001	1.700	10.731	0.000	0.000	/
TCONS_00071370	0.001	0.167	7.381	0.000	0.000	/
TCONS_00061798	0.003	1.347	8.658	0.000	0.020	PRKCA
TCONS_00079817	0.001	0.673	9.395	0.000	0.000	/
TCONS_00109437	0.383	0.001	−8.582	0.000	0.022	/

### Differentially expresgessed miRNAs and circRNAs during intramuscular fat deposition

**Fig. 4:**
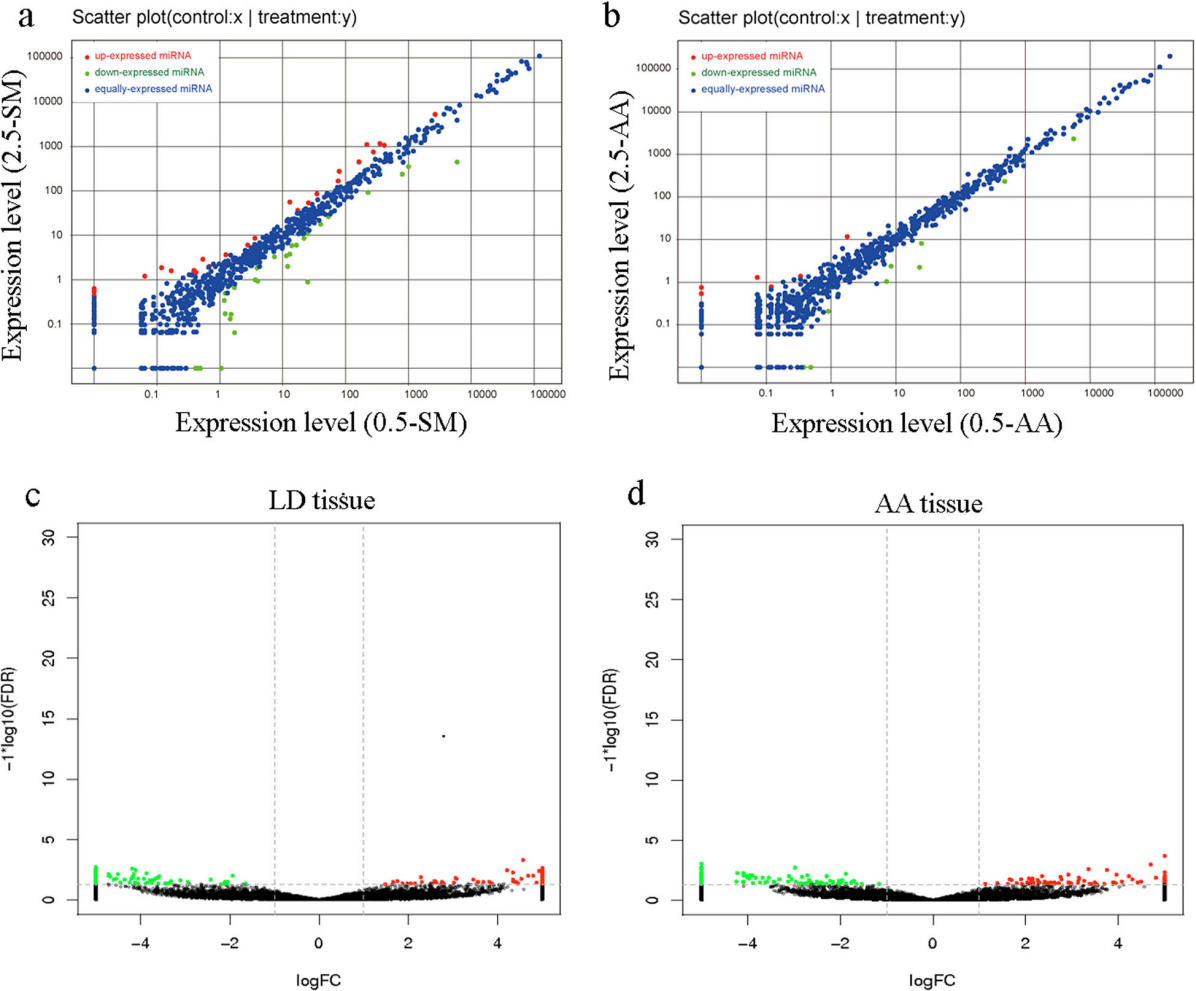
Differentially expressed miRNAs and circRNAs during LD and AA development, respectively. (**a** and **b**) differentially expressed miRNAs. (**c** and **d**) differentially expressed circRNAs. (**a** and **c**) LD tissue. (**b** and **d**) AA tissue

In total, 62 differentially expressed miRNAs (DEMs) were obtained in LD tissues, where 30 were upregulated and 32 were downregulated (Fig. 4a, Table S7). KEGG pathway analysis revealed that these DEMs were significantly enriched in 94 pathways, some of which were important for lipid biosynthesis; for example, the PI3K-Akt signaling, MAPK signaling, AMPK signaling, fatty acid metabolism, and biosynthesis of unsaturated fatty acids pathways. Moreover, 15 DEMs were obtained in the AA tissues, of which 6 were upregulated and 9 were downregulated (Fig. 4b, Table S7). The targets of these 15 DEMs were significantly enriched in 63 pathways, some of which were related to lipid metabolism; for example, the Hippo signaling, MAPK signaling, AMPK signaling, and PI3K-Akt signaling pathways (Table S7). Furthermore, two miRNAs (miR-122-x and miR-381-y) were simultaneously downregulated in both the AA and LD tissues, and one novel miRNA (novel-m0085-5p) was contemporaneously upregulated in both tissues. Two miRNAs (miR-208-y and miR-499-y) exhibited opposite expression trends, being upregulated in LD tissue but downregulated in AA tissue (Table 4).

**Table 4:** The co-differentially expressed miRNAs in LD and AA

ID	Differentially expressed miRNAs in LD				Differentially expressed miRNAs in AA			
	0.5-LD	2.5-LD	log2(FC)	*P*-Value	0.5-AA	2.5-AA	log2(FC)	*P*-Value
miR-122-x	24.998	0.889	−4.813	0.000	23.257	2.242	−3.375	0.024
miR-208-y	413.741	1073.040	1.375	0.000	7.216	1.044	−2.790	0.010
miR-381-y	5959.075	448.340	−3.732	0.000	480.054	231.358	−1.053	0.047
miR-499-y	352.348	1168.921	1.730	0.000	8.458	2.389	− 1.824	0.041
novel-m0085-5p	0.010	0.632	5.982	0.017	0.010	0.754	6.237	0.016

We also identified 223 differentially expressed circRNAs (DECs; 125 upregulated and 98 downregulated) in the LD tissue (Fig. 4c, Table S8). KEGG pathway analysis revealed that these DECs were significantly enriched in the cyclic guanosine monophosphate (cGMP)–protein kinase G (PKG) signaling pathway, and involved in pathways related to lipid and carbohydrate metabolism; for example, the propanoate and pyruvate metabolism, fatty acid biosynthesis, Hippo signaling, and MAPK signaling pathways (Table S8). Moreover, 211 DECs (91 upregulated and 120 downregulated) were obtained in the AA tissues (Fig. 4d, Table S8), where function annotation results revealed that they were enriched in pathways related to lipid metabolism, such as the AMPK signaling, fatty acid biosynthesis, and fatty acid metabolism (Table S8). Of these DECs in the LD and AA tissues, *circRNA000230* and *circRNA053707* were found to be simultaneously downregulated, whereas *circRNA008790* and *circRNA040844* were simultaneously upregulated. In addition, *circRNA054960* was upregulated in the LD tissue but downregulated in the AA tissue (Table 5, Table S8).

**Table 5:** The co-differentially expressed circRNAs in LD and AA

ID	Differentially expressed circRNAs in LD				Differentially expressed circRNAs in AA			
	0.5-LD	2.5-LD	log2(FC)	*P*-value	0.5-AA	2.5-AA	log2(FC)	*P*-value
novel_circ_000230	51.640	0.001	−15.656	0.030	118.207	0.001	−16.851	0.014
novel_circ_008790	0.001	46.645	15.509	0.031	4.372	113.617	4.700	0.001
novel_circ_040844	5.920	119.726	4.338	0.005	8.588	82.063	3.256	0.025
novel_circ_053707	143.682	0.001	−17.133	0.034	393.484	0.001	−18.586	0.006
novel_circ_054960	3.137	80.385	4.679	0.011	114.972	23.343	−2.300	0.046

### Construction of the ceRNA coregulatory network

**Fig. 5:**
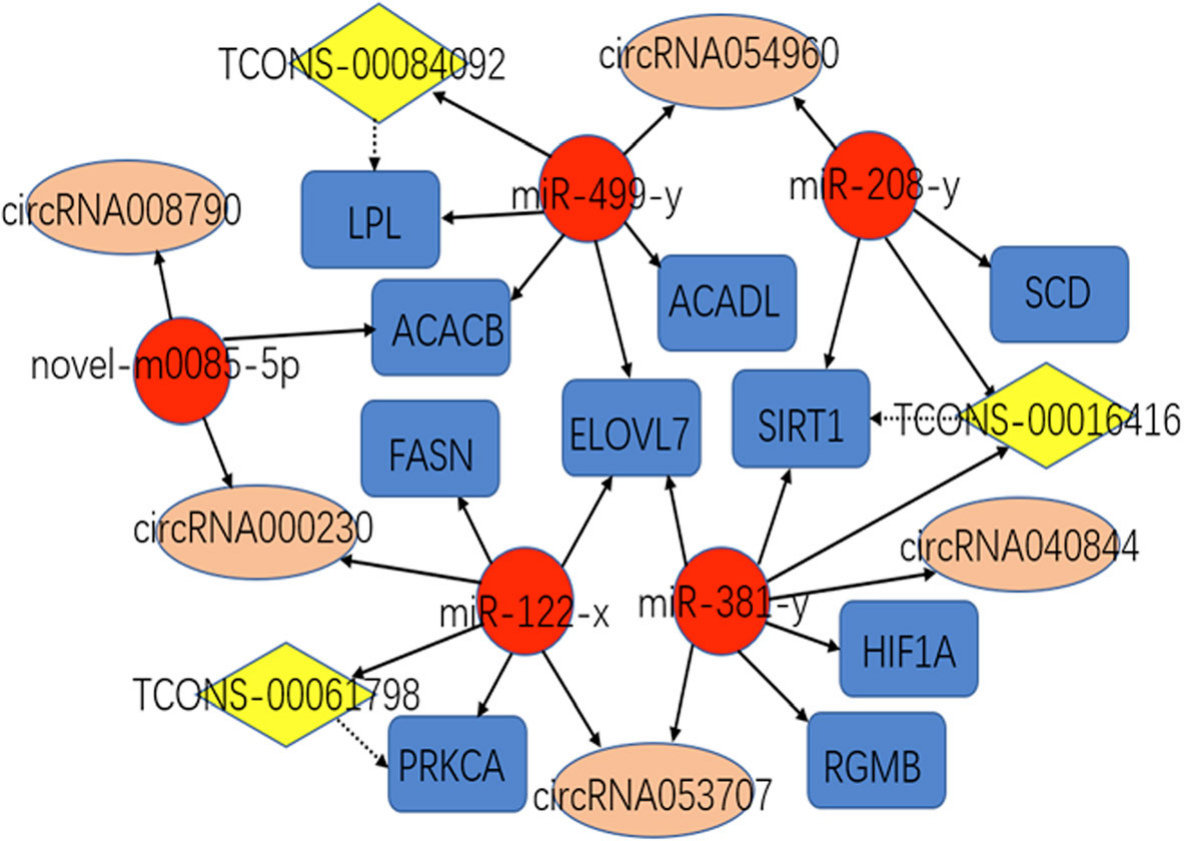
The ceRNA co-regulation network. The ceRNA co-regulation network. Co-differentially expressed mRNAs, miRNAs, lncRNAs, and circRNAs during AA and LD tissues development were used to construct the ceRNA co-regulation network. The bule box, red circle, yellow rhombus, and pink ellipse nodes represent co-differentially expressed mRNAs, miRNAs, lncRNAs, and circRNAs, respectively. The dotted line and the solid line indicate the co-regulation between lncRNAs and mRNAs, and between miRNAs and other transcripts, respectively

It has been shown that mRNAs, lncRNAs, and circRNAs may act as ceRNAs, which regulate gene function via miRNA in various processes [22, 23], suggesting that ceRNAs and their miRNAs may be coregulated in IMF deposition. On the basis of the data of the co-differentially expressed mRNA, lncRNA, circRNA, and miRNA transcripts, we obtained the mRNA-miRNA, lncRNA-miRNA, and circRNA-miRNA pairs, combined them with the lncRNA-mRNA pairs, and then constructed the integrated ceRNA network. The constructed network contained 10 DEGs, 5 DEMs, 5 DECs, 3 DELs, and 29 relationships (Fig. 5). Within the network, it was found that both *TCONS-00016416* and its target *SIRT1* could be targeted by miR-381-y. the same results were observed for the miR-122-x-*TCONS-00061798-PRKCA* and miR-499-y-*TCONS-00084092-LPL* ceRNA subnetworks, suggesting that *SIRT1*, *PRKCA* and *LPL* may be the crucial genes mediated by noncoding RNAs for regulating IMF deposition.

### RT-qPCR validation of gene expression

**Fig. 6:**
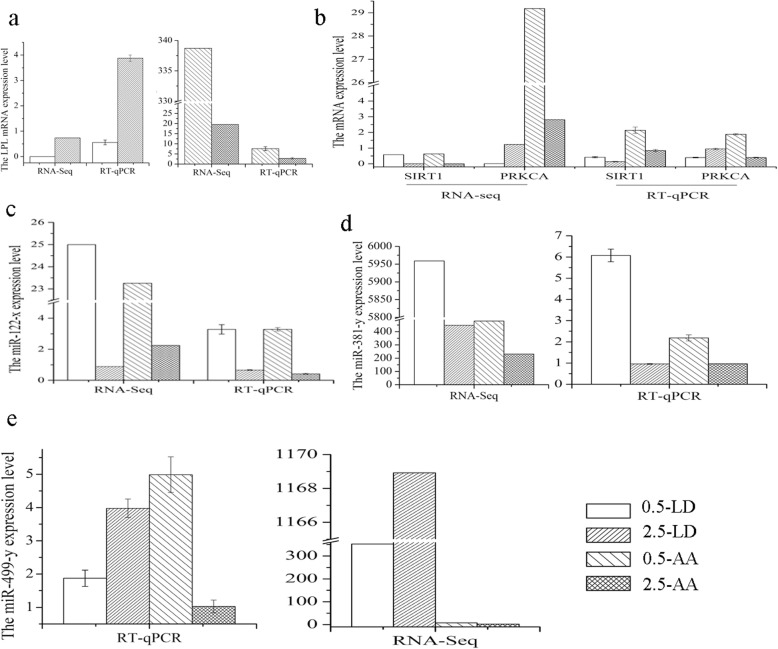
The validation of the mRNA and miRNA RNA-seq results. (**a**) the LPL mRNA expression level. (**b**) the SIRT1 and PRKCA mRNA expression level. (**c** to **e**) the miR-122-x(**c**), miR-381-y(**d**) and miR-499-y(**e**) expression level. 0.5-LD: 0.5-year-old *longissimus dorsi* muscle tissues. 2.5-LD: 2.5-year-old *longissimus dorsi* muscle tissues. 0.5-AA: 0.5-year-old adjacent adipose tissue. 2.5-AA: 2.5-year-old adjacent adipose tissue. RNA-seq: the results of RNA-seq. RT-qPCR: the results of RT-qPCR

**Fig. 7:**
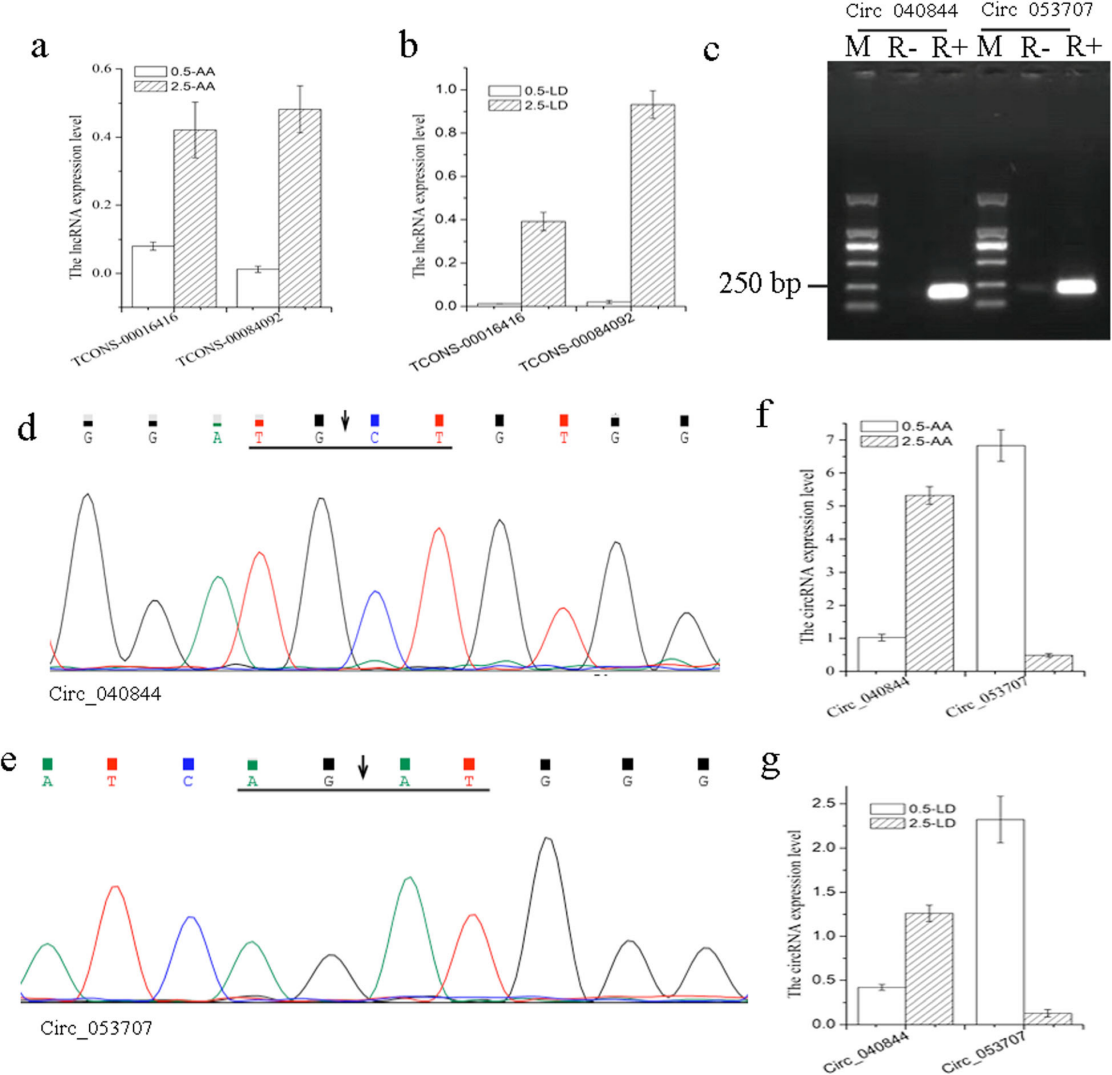
The validation of the lncRNA and circRNA RNA-seq results. (**a** and **b**) the validation of the lncRNAs expression in LD and AA tissues. (**c**) Circ_040844 and Circ_053707 were amplified with RNase R-digested RNA or undigested RNA as templates. M. DL2000 DNA marker; “R+” indicates RNA treated with RNase R; “R-” indicates untreated RNA. (**d** and **e**) The Sanger sequencing of Circ_040844 and Circ_053707 RT-PCR products. The black arrows indicate head-to-tail back-splicing sites of circRNAs. (**f** and **g)**. the validation of the circRNAs expression in LD and AA tissues. 0.5-LD: 0.5-year-old *longissimus dorsi* muscle tissues. 2.5-LD: 2.5-year-old *longissimus dorsi* muscle tissues. 0.5-AA: 0.5-year-old adjacent adipose tissue. 2.5-AA: 2.5-year-old adjacent adipose tissue

Validation of the RNA-seq results was carried out using the quantitative reverse-transcription polymerase chain reaction (RT-qPCR) for 3 DEGs (*LPL*, *SIRT1* and *PRKCA*), 3 DEMs (miR-122-x, miR-381-y, and miR-499-y), 2 DELs (*TCONS-00016416*,and *TCONS-00084092*), and 2 DECs (Circ_040844, and Circ_053707). The expression of these selected transcripts was significantly different in both the LD and AA tissues during yak development, with the expression patterns being highly consistent with those obtained by the RNA-Seq method (Figs. 6 and 7). It’s worth mentioning that the backsplice junction of circRNA were confirmed before the validation, as shown in Fig. 7, due to the circular structure, the circRNA is more resistant to digestion by RNase R treatment, and the back-splicing sites were verified by Sanger sequencing (Fig. 7d and e). The results indicated the high reproducibility and reliability of the gene expression profiles obtained in our study.

## Discussion

As is widely known, the IMF content is one of the polygenic traits in animal that is an important determinant of meat quality characteristics. Given the importance of IMF on the economics of livestock production, the clarification of the molecular mechanisms underlying IMF deposition holds great significance. The associations of genomic markers with IMF deposition are not always consistent depending on the species or the breeds. To our best knowledge, this is the first study that has comprehensively analyzed the whole-transcriptome profiles related to yak meat characteristics. In this study, to identify genes that are related to IMF deposition in the yak, we first measured the IMF content in LD of 0.5-, 2.5-, 4.5-, and 7.5-year-old yaks, whereupon it was found that the IMF content was deposited quickly from 0.5 to 2.5 years. This prompted us to choose LD and AA tissues in these two developmental stages for the whole-transcriptome profile analysis.

In total, 149 and 72 DEGs were identified during yak muscle and adipose tissue development, respectively. Of these, 16 DEGs were co-differentially expressed in both tissues, many of which had known functions in lipid metabolism. For instance, the lipogenesis genes *ACACB*, *FASN*, *SCD*, and are involved in the fatty-acyl-CoA biosynthetic process and play catalytic roles in fatty acid biosynthesis [24, 25]. LPL is the principal enzyme that acts as a key factor in the hydrolysis of triacylglycerol and uptake of free fatty acids from the plasma. ACADL, one of the rate-limiting enzymes in fatty acid beta-oxidation, has been identified as candidate function gene in IMF deposition in chicken [26]. Moreover, the expression dynamics of *AUST2*, *ACADL*, *ELOVL7*, *FASN*, *ZNF41*, and *SCD* during muscle and adipose development are in consistent with the increased trend of IMF deposition, whereas SIRT1 showed the opposite trend, indicating that the former genes act as positive regulators in IMF deposition whereas the latter gene acts as negative regulator in this process. Interestingly, *XAB2*, *RGMB*, *SMAD1*, *PRKCA*, *MAP 4 K1*, *LPL*, and *HIF1A* upregulated during muscle development, but downregulated during adipose tissue development, whereas *ACACB* and *GNA12* showed the opposite expression trend. These consistent or inconsistent trend changes in expression trends may be related to the different function of these genes during the development of these two tissues, which are worthy of future analysis.

As a major class of the noncoding RNAs, lncRNAs could act as key regulators in many biological and pathological processes via *trans*, cis, and antisense activities. PU.1 expression was found to be modulated by an antisense lncRNA that was transcribed from the PU.1 gene itself in immune-related cell lines [27] and preadipocytes [28]. The lncRNA *Jpx* acts both in *trans* and in *cis* to activate X specific transcripts expression in mouse embryonic cells [29]. Thus, the prediction of lncRNA target genes through these three independent algorithms together with the KEGG analysis would be useful for identifying which processes lncRNAs are involved in and further revealing their potential functions. Our results showed that lncRNAs obtained in this study may regulate pathways related to lipid or carbohydrate metabolism (e.g., steroid hormone biosynthesis, hedgebog signaling, and glycolysis and gluconeogenesis pathways) via *trans*, *cis*, and antisense activities. Previously studies have shown that the Hedgebog pathway enables cells to sense and respond to Hedgebog ligands, which are covalently modified by cholesterol, indicating that there is a connection between Hedgehog signaling and lipid metabolism [30]. Furthermore, our results identified 4 DELs and 9 DELs during muscle and adipose development, respectively, and found that 3 co-DELs may regulate 3 lipid-related genes (*LPL*, *SIRT1* and *PRKCA*) via antisense and *trans* activities. As an enzyme, LPL is involved in fatty acid catabolism, and its expression level is positively associated with IMF content [31]. Moreover, several lines of evidence have demonstrated that SIRT1(a key metabolic/energy sensor) plays an important role in regulating lipid metabolism by deacetylating some transcriptional regulators and co-activators, for instance, carbohydrate response element binding protein (ChREBP), sterol regulatory element binding protein-1c (SREBP-1c), PPARα, nuclear factor-κB (NF-κB), ect [32].. On the basis of these results, we suspected that one of the main roles of these 3 lncRNAs is to regulate the IMF deposition in yak, and further highlighting of their detailed mechanisms in this process would be fertile ground for future investigation.

The results on the DEMs and DECs during muscle and adipose tissue development indicated that they were significantly enriched in lipid-related pathways, for instance, MAPK signaling [33, 34], PI3K-Akt signaling [35], AMPK signaling [36, 37], fatty acid biosynthesis, fatty acid metabolism pathways. AMPK represses fatty acid, triglyceride, and cholesterol synthesis through several ways, including through acetyl-CoA carboxylase [36], 3-hydroxy-3-methylglutaryl-coenzyme A reductase (HMGCR), and SREBP2 phosphorylation, and represses the proteolytic processing, nuclear translocation, and transcriptional activity of SREBP1 [37]. Interestingly, 5 DEMs and 5 DECs were found to be co-differentially expressed during muscle and adipose tissue development. Although the DECs are novel circRNAs that were not previously identified, very little information or data is available from published databases, it was reported that these DECs are involved in lipid metabolism, such as miR-122 was discovered to be involved in the regulation of cholesterol and fatty acid homeostasis in human and mice [38, 39]; miR-499 can negatively regulate the PR domain containing 16 expression and hinder skeletal muscle satellite cells adipogenic differentiation [40].

Combined with the co-differentially expressed DEGs, DELs, DECs, and DEMs, we constructed a ceRNA regulatory network, which showed that 10 DEGs, 5 DECs, and 3 DELs cross-talked with one another through the 5 DEMs. This indicated that IMF deposition in yak results from a balance level of gene expression, and that the development of IMF is a complex multi-organ process regulated by the coordinated actions of muscle and adipocyte tissues. Furthermore, from the ceRNA network, we observed three ceRNA subnetworks, which showed that *TCONS-00016416* and its target *SIRT1* “talked” to each other through the same miR-381-y and miR-208-y response elements, whereas *TCONS-00061798* and its target PRKCA, and *TCONS-00084092* and its target *LPL*, “talked” to each other through miR-122-x and miR-499-y response elements, respectively. Therefore, we speculate that these three subnetworks may play a key role in the regulation of IMF deposition.

Although we successfully found many DEGs that may be associated with IMF content and constructed a ceRNA regulatory network for the yak, some limitations in this study should be noted. Since the collection of IMF in yak is difficult, we sampled the LD and AA tissues to synthetically analyze the potential IMF-related genes to overcome the limitations of IMF sampling, which may to some extent have affected the results and explanations. Nonetheless, the genes involved in the ceRNA regulatory network may play an important role in IMF deposition through molecular synergism and upregulation of important pathways, these possibilities are worthy of future research efforts.

## Conclusions

The present study provides a comprehensive landscape of the differences in the whole- transcriptome profiles of LD and AA tissues between two developmental stages in yaks. We identified 16 DEGs related to lipid biosynthesis that were co-differentially expressed in the two tissues during development, including *ACACB*, *ACADL*, *ELOVL7*, *SIRT1*, *FASN*, *LPL*, *PRKCA*, and *SCD*. Furthermore, we found that several differentially expressed lncRNAs, miRNAs, and circRNAs during muscle and adipose development were closely related to some lipid metabolism pathways, and that the 3 lncRNAs, 5 miRNAs, and 5 circRNAs that were co-differentially expressed in the two tissues may play a crucial role in regulating IMF deposition. On the basis of the co-differently expressed transcripts, we constructed a ceRNA regulatory network which contained 10 DEGs, 5 DEMs, 5 DECs, 3 DELs, and 29 relationships. Within the network, we suspected that the 3 ceRNA subnetworks (i.e., miR-381-y-*TCONS-00016416*-*SIRT1*, miR-122-x-*TCONS-00061798*-*PRKCA*, and miR-499-y-*TCONS-00084092*-*LPL*) may play a crucial role in the regulation of IMF deposition. Our findings have identified potential regulators and molecular regulatory networks that may be involved in IMF contents in yaks, and provide a foundation for future studies on the molecular mechanisms underlying IMF deposition.

## Methods

### Animals and sample collection

In total, 12 LWQ female yaks that had been raised in grazing systems and under the same conditions of handing and nutrition in natural pasture of Leiwoqi country (Location: Changdu, Tibet, China; geographic coordinates: 96°23′33″E, 31°27′3″N, altitude:4200 m above sea level) were randomly selected at four developmental stages (0.5, 2.5, 4.5, and 7.5 years of age). Each stage comprised three yaks with the similar body weight. Between Oct 21st and 22 nd, 2017, all yaks were stunned with a captive bolt pistol (Cash 8000 Model Stunner, 0.22 calibre, 4.5 grain cartridge) to ameliorate the suffering of the animals prior to their humane killing, following which exsanguination via a transverse incision of the neck was carried out in the slaughterhouse of Zang Jia Mao Niu Co, Ltd. Then, the LD and AA tissues were excised immediately between the 12th and 13th ribs (right half carcass) and rapidly stored in liquid nitrogen until RNA isolation. We also collected one more LD sample for IMF content measurement. All animals used in this study belong to Zang Jia Mao Niu Co, Ltd.

### Analysis of the intramuscular fat content

The IMF content of the 12 LD samples were determined according to the standard Soxhlet extraction method [41, 42]. In brief, the LD sample was pre-dried and crushed following weighted an x amount (in grams) into the Soxhlet glass tube, and then transferred to the extraction chamber in the Soxhlet equipment. The sample was soaked overnight in anhydrous ether, following which the anhydrous ether backflow devices were opened for 10 h at 80 °C. The residue sample was dried under a fume hood for1 h and then transferred to a forced-air oven at 105 °C for 8 h. The dried residue sample was weighted and marked as the y amount (in grams). The IMF content was calculate as follows: IMF(%) = [(x-y)/x] × 100.

### Total RNA isolation, sequencing and raw data analysis

On the basis of the IMF content results, total RNA was isolated from LD and AA samples excised from the 0.5- and 2.5-year-old yak with TRIzol reagent (Invitrogen, CA, USA). Then, DNase and an RNeasy Mini Kit (Qiagen, CA, USA) were used to purify the total RNA. NanoDrop 2000 Spectrophotometer (Thermo Fisher Scientific, DE, USA), Bio-Photometer (Eppendorf, Hamburg, Germany) and 1% agarose gel electrophoresis were used to measure the quantity and quality of the extraction total RNA. Furthermore, the RNA Nano 6000 Assay Kit with the Agilent Bioanalyzer 2100 system (Agilent Technologies, CA, USA) were used to assess the RNA integrity.

After removed the ribosomal RNA with the Ribo-Zero rRNA kit (Epicentre, WI, USA), the RNA libraries (mRNAs, lncRNAs, and circRNAs) were generated with the mRNA-Seq Sample Preparation Kit (Illumina, CA, USA). The library quality was measured with the Agilent Bioanalyzer 2100 system and then sequenced using the Illumina HiSeq™ 4000 system according to the vendor’s recommended protocol. Those containing ploy-N or adapter and low quality reads were removed from the sequenced raw reads, the retained reads were named clean reads. The Tophat2 software with default parameters was used to map the clean reads to the *Bos grunniens* genome (BosGru v2.0), and the mapped reads of each sample that existed at least one of both replicates were assembled with StringTie software using the default parameter. The annotated and unannotated transcripts were obtained using Cufflinks after reconstruction of the transcripts from our RNA-seq data. The coding potential calculator, Coding-Non-Coding-Index (CNCI, version 2) and Pfam Scan were used with default parameter to predict the annotated transcripts coding potential. Those transcripts that were predicted to have coding potential by two or all of the above three tools named as candidate set of novel protein-coding transcripts, whereas those without coding potential were named as novel lncRNAs. The different types of lncRNAs (inclusive of *cis*-acting, antisense, and *trans*-acting) were selected using Cuffcompare. The circRNA Identifier (CIRI) tool was used to identify the circRNAs according to previously studies [43, 44]. The DECs were identified using EBSeq.

Refer to the standard procedure, miRNA libraries were constructed and then the quality was assessed as above cDNA libraries. The libraries were then sequenced with Illumina HiSeq™ 2500 system according to the vendor’s recommended protocols. Clean reads were obtained after the removal of raw reads containing 5′ adaptor, 3′ adaptor, no insertion sequence, and poly(A) in small RNA fragments, as well as those shorter than 18 nt, of known bovine classes of RNAs (ribosomal RNAs, messenger RNAs, small nuclear RNAs, transfer RNAs, small nucleolar RNA, small cytoplasmic RNAs, and repeats), and of low quality. The retained reads were mapped to the miRNAs in the miRBase 22.0 database (http://www.mirbase.org/), the mapped one was named as known miRNA. While, the unmapped one was then aligned to the yak genome, and the mirdeep2 algorithm was used to predict novel miRNA. Three softwares (mireap, miRanda and TargetScan) with default parameters were used to predict miRNAs and circRNAs targets.

All transcripts expression level was calculated using the Stringtie and Ballgown tools, and normalized using FPKM with the RSEM software. The false discovery rate (FDR) and FC were analyzed using the edgeR package, only the transcripts with a FC of ≥2 and FDR < 0.05 were then assigned as differentially expressed transcripts.

### Gene ontology enrichment and KEGG pathway analyses

GO annotation and KEGG enrichment analyses were conducted to annotate the potential function of the genes. GO enrichment analysis was carried out with the GOseq R package, and the GO terms of the DEGs were assessed using Fisher’s exact test. The DAVID web server annotation tool (version 6.8, http://david.ncifcrf.gov/) was used to map the enriched pathways from the KEGG database. Only the *p* < 0.05 was considered statistically significant and listed.

### Prediction of lncRNA and miRNA targets and construction of the ceRNA network

Previous studies hypothesized that mRNAs, lncRNA, and transcribed pseudogenes “talk” to one another through miRNA response elements [22], which form a large number of complex regulatory networks and play an important role in various biological processes. On the basis of this theory, we constructed a co-expression ceRNA network related to the regulation of IMF deposition in the yak. The detail methods are as given below:

Three strategies: *cis-acting*, antisense, and *trans-acting* regulation prediction, were used to predict the targets of lncRNAs. For the *cis*-acting prediction, the locations of the paired lncRNAs and mRNAs in the genomic of yak were calculated, and the genes within 10 kb of the lncRNAs were named as *cis*-acting regulatory targets. For antisense regulation prediction, the RNAplex software were used to screen targets by comparing complementary bases between the lncRNAs and mRNAs. For *trans*-acting regulatory targets, the expression of the lncRNA was determined to be not related with the location of the mRNA but co-expressed with it. To predict the lncRNA targets, the free energy between them was calculate using LncTar.

The miRcode web server (version 11, http://www.mircode.org/) was used for predicting the lncRNA-miRNA interactions, whereas TarBase (version 7.0, http://biogps.org/plugin/1225/diana-tarbase-v70/), miRanda (version 3.3a, http://www.miranda.org/) and TargetScan (http://www.targetscan.org/vert_72/) were used for predicting the miRNA-mRNA interactions.

The co-expression of DECs and DEMs were measured with pearson’s correlation coefficient method, where the absolute coefficient value that greater than 0.8 was considered relevant for the network construction, with *p* < 0.05 regarded as being statistically significant. Based on the correlation analysis between DECs and DEMs, The co-expression network of circRNA-miRNA was constructed.

Upon these analyses above, we selected the co-differentially expressed DEGs, DELs, DECs, and DEMs involved in these co-expression networks to construct the co-expression ceRNA regulatory network related to IMF-deposition.

### RNA sequencing results validation using RT-qPCR

To evaluate the reliability of the transcript expression data obtained by RNA-Seq. RT-qPCR were carried out with the LD and AA tissues. Using the PrimeScript™ RT reagent Kit (Takara, Dalian, China), total RNA was reverse transcribed for mRNA and miRNA detection. Using M-MLV reverse transcriptase kit (Takara, Japan) and random primers, total RNA was reverse transcribed for lncRNA evaluation. To verify the back-splicing junction of circRNA, the RNase R- and control reaction (without RNase R) were prepared and reverse transcribed into cDNA to amplify each circRNA with primers following previously method [45, 46], then the products were sequenced to find the back-splicing sites. To experimentally evaluate the expression of circRNAs in LD and AA tissues, RNase R-treated cDNA were used as PCR templates. All qPCR experiments were conducted using the SYBR Premix Ex Taq kit (Takara, Dalian, China). The *GAPDH*, β-actin, and U6 small nuclear RNA genes were selected as the endogenous control genes (all primers are shown in Table S9). All qPCR validations were carried out with three biological replicates and triplicate reactions for each sample. After amplification, the products were confirmed by agarose gel electrophoresis and Sanger sequencing, the relative transcript abundance was calculated using 2^-ΔΔCt^ method.

## Supplementary information

**Additional file 1: Table S1.** Overview of the data for RNA sequencing. **Table S2.** Overview of the data for small RNA sequencing.

**Additional file 2: Table S3.** Identified mRNAs, lncRNAs, circRNAs, and miRNAs in our data.

**Additional file 3: Table S4.** Identified tissue-specific expressed mRNAs, lncRNAs, and circRNAs in our data.

**Additional file 4: Table S5.** Differentially expressed genes during LD and AA development, and GO enrichment and KEGG pathway analysis for differential expression genes.

**Additional file 5: Table S6.** The targets prediction of lncRNAs and the differentially expressed lncRNAs during LD and AA development.

**Additional file 6: Table S7.** Differentially expressed miRNAs during LD and AA development, and KEGG pathway analysis for their target genes.

**Additional file 7: Table S8.** Differentially expressed circRNAs during LD and AA development, and KEGG pathway analysis for their target genes.

**Additional file 8: Table S9.** Primer sequences for RT-qPCR.

## Data Availability

The data supporting the conclusions of this article can be found in Tables S3, S4, S5, S6 ,S7, S8, S9. The RNA sequencing data generated during the current study has been uploaded in the NCBI BioSample database (https://www.ncbi.nlm.nih.gov/biosample/?term). The accession numbers of RNA-seq data of the 12 samples are SAMN14599609, SAMN14599610, SAMN14599611, SAMN14599612, SAMN14599613, SAMN14599614, SAMN14599615, SAMN14599616, SAMN14599617, SAMN14599618, SAMN14599619, SAMN14599620, respectively. The accession number for RNA-seq data deposited in NCBI BioProject is PRJNA624986. The *Bos grunniens* genome was obtained from NCBI using the accession number: BosGru v2.0.

## Abbreviations

AA: Adjacent adipose
ACADL: Long-chain acetyl-coenzyme A dehydrogenase
ceRNA: Competing endogenous ribonucleic acid
circRNA: Circular ribonucleic acid
DECs: Differentially expressed circular ribonucleic acids
DEGs: Differentially expressed genes
DELs: Differentially expressed long noncoding ribonucleic acids
DEMs: Differentially expressed micro-ribonucleic acids
FASN: Fatty acid synthase
GO: Gene Ontology
IMF: Intramuscular fat
KEGG: Kyoto Encyclopedia of Genes and Genomes
lncRNA: Long noncoding ribonucleic acid
LPL: Lipoprotein lipase
miRNA: Micro-ribonucleic acid
PRKCA: Protein kinase C alpha
SCD: Stearoyl-CoA desaturase
SIRT1: Sirtuin 1
LD: *Longissimus drosi* muscle
